# Increase of theta frequency is associated with reduction in regional cerebral blood flow only in subjects with mild cognitive impairment with higher upper alpha/low alpha EEG frequency power ratio

**DOI:** 10.3389/fnbeh.2013.00188

**Published:** 2013-12-05

**Authors:** Davide V. Moretti, Annapaola Prestia, Giuliano Binetti, Orazio Zanetti, Giovanni B. Frisoni

**Affiliations:** Alzheimer Unit, IRCCS San Giovanni di Dio FatebenefratelliBrescia, Italy

**Keywords:** EEG, alpha3/alpha2 frequency ratio, SPECT, mild cognitive impairment, Alzheimer's disease

## Abstract

**Background**: Several biomarkers have been proposed for detecting Alzheimer's disease (AD) in its earliest stages, that is, in the predementia stage. In an attempt to find noninvasive biomarkers, researchers have investigated the feasibility of neuroimaging tools, such as MRI, SPECT as well as neurophysiological measurements using EEG. Moreover, the increase of EEG alpha3/alpha2 frequency power ratio has been associated with AD-converters subjects with mild cognitive impairment (MCI).

**Objective**: To study the association of alpha3/alpha2 frequency power ratio with regional cerebral blood flow (rCBF) changes in subjects with MCI.

**Methods**: Twenty-seven adult subjects with MCI underwent EEG recording and perfusion single-photon emission computed tomography (SPECT) evaluation. The alpha3/alpha2 frequency power ratio was computed for each subject. Two groups were obtained according to the median values of alpha3/alpha2, at a cut-off of 1.17. Correlation between brain perfusion and EEG markers were detected.

**Results**: Subjects with higher alpha3/alpha2 frequency power ratio showed a constant trend to a lower perfusion than low alpha3/alpha2 group. The two groups were significantly different as about the hippocampal volume and correlation with the theta frequency activity.

**Conclusion**: There is a complex interplay between cerebral blood flow, theta frequency activity, and hippocampal volume in MCI patients with prodromal Alzheimer's disease, characterized by higher EEG alpha3/alpha2 frequency power ratio.

## Introduction

The identification and validation of biomarkers for diagnosing, monitoring progression, and predicting onset of Alzheimer's disesae (AD) has been a main focus of AD research in the past 10 years. In line with recently published research criteria., it is becoming clear that the integration of different biomarkers is a milestone for a correct and early diagnosis of mild cognitive impairment (MCI) due to AD (Dubois et al., 2007; Albert et al., 2011). To date, the most studied and validated biomarkers are Abeta42 and Tau protein in the cerebrospinal fluid (CSF), glucose metabolism on fluorodeoxyglucose positron emission tomography (18F-FDG PET), regional cerebral blood flow (rCBF) on single photon computed emission tomography (SPECT), atrophy of hippocampal volume (HV) on magnetic resonance imaging (MRI), and brain amyloid deposition on amyloid imaging with PET (Hampel et al., 2008; Galluzzi et al., 2013). Anyway, some controversies remain to debate. The latter biomarkers have a good sensibility in identifying subjects with a neurodegenerative disorders at high risk to convert in dementia, but they lack a reliable specificity that allow a clear-cut diagnosis of the different subtypes of dementias. Of note, in neurodegenerative disorders, like AD or other dementias, the brain networks modifies many years before clinical manifestations.

Recent SPECT studies have demonstrated that a large neural network is altered in subjects with prodromal AD, including precuneus, medial temporal, parietal ad frontal cortices (Launes et al., 1991; Rodriguez et al., 1999, 2004; Frisoni et al., 2003; Gungor et al., 2005). For instance, selective rCBF reductions in the left hippocampus and parahippocampal gyrus and in extended areas of cerebral association cortex were demonstrated in a 2 years follow-up clinical study with rCBF-SPECT (Kogure et al., 2000; Pupi et al., 2005). Cross-sectional studies have shown rCBF and regional metabolic rates of glusocse (rCMRgl) reductions in the resting state throughout the cortex in AD, involving distinctive brain structures such as the posterior cingulate/precuneus, temporoparietal, and frontal cortices (Minoshima et al., 1994). A positive SPECT scan raised the likelihood of diagnosing pathological AD from 84%, as defined by clinical diagnosis, to 92% (Jagust et al., 2001; Frisoni, 2012). Recent results show that there is a hippocampal rCBF hypoperfusion in patients with mild AD (Rodriguez et al., 2000), as well as that baseline SPECT can support outcome prediction in subjects with MCI (Nobili et al., 2009). Of note, rCBF (bilateral parietal perfusion) and qEEG (especially the slowest frequencies, i.e., 2–5.5 Hz) are confirmed to be good descriptors of AD severity. It is especially noteworthy that bilateral hippocampal rCBF reduction was the perfusional index best correlated with both cognitive performance and qEEG (Rodriguez et al., 1999, 2004).

In the conceptual frame of the integration of biomarkers for an early and highly predictive diagnosis, the EEG could be a reliable tool (Moretti et al., 2003, 2004, 2007a,b, 2008a,b, 2009a,b,c, 2011, 2012, 2013a,b; Missonnier et al., 2010). Indeed, it is widely accepted that the cerebral EEG rhythms reflect the underlying brain network activity (Steriade et al., 1990; Steriade, 2006). As a consequence, modifications in EEG rhythms could be an early sign of disease associated with AD-related structural and functional networks. Recently, it has been demonstrated that the increase of high alpha relative to low alpha frequency power is a reliable EEG marker of hippocampal atrophy (Moretti et al., 2009a) and amigdalo-hippocampal complex atrophy (Moretti et al., 2009b). Furthermore, the increase in alpha3/alpha2 power ratio has been demonstrated predictive of conversion of patients with MCI in AD, but not in non-AD dementia (Moretti et al., 2011, 2013a,b). The same increase of alpha3/alpha2 frequency power ratio was found to be correlated with hippocampal atrophy in subjects with AD (Moretti et al., 2012). Finally, a recent study have shown that MCI subjects with highest alpha3/alpha2 frequency power ratio present a peculiar pattern of basal ganglia and thalamic atrophy, detected with voxel-based-morphometry (VBM) tecnique, as compared to MCI groups with middle and low alpha3/alpha 2 power ratio (Moretti et al., 2012, 2013a,b). The present explorative study shares the same theoretical background. It could be of remarkable scientific interest to confirm that alpha3/alpha2 power ratio is a reliable diagnostic marker extending his relationship with rCBF detected with SPECT. It is not a simple association study between EEG and rCBF, because the individuated cut-off for the alpha3/alpha2 frequency power ratio makes it available to be used in the single patient and not only at level group, as happens in most other studies. As a consequence, the working hypothesis of the present study is that an increase in alpha3/alpha2 EEG frequency power ratio would like to be associated with lower brain perfusion in specific brain areas. To the best of our knowledge, it is the first study that investigates the association between a specific EEG marker, which has been demonstrated promising in detecting prodromal AD, and the rCBF.

## Methods

### Subjects

#### MCI patients

MCI patients were taken from a prospective project on MCI (“Mild Cognitive Impairment in Brescia—MCIBs”), aimed to study the natural history of persons without dementia with apparently primary cognitive deficits, i.e., not due to psychic or physical conditions. The study protocol was approved by the local ethics committee and all participants signed an informed participation consent. Details on inclusion/exclusion criteria and on physical and neurological examinations, performance-based tests of physical function, gait and balance and performed neuropsychological battery have been previously published and are at disposable elsewhere (Caroli et al., 2007a). Among the 56 MCI patients who agreed to undergo MRI and SPECT scan, all consecutive 27 who agreed also to undergo EEG recording were further considered.

#### Normal controls

We enrolled all 17 healthy subjects from a previous study on cerebral perfusion correlates of conversion to AD with both an MRI and a SPECT scan available (for inclusion/exclusion criteria and for a detailed description of the enrolment procedure please see Caroli et al., 2007a). Briefly, subjects were consecutive normal volunteers picked among those undergoing brain MRI scan at the Neuroradiology Unit of the “Città di Brescia”Hospital in Brescia from October 2004 to June 2006 for reasons unrelated to cognition, or were healthy volunteers aged 65 years or older, among MCI patients' spouses, friends of them, and researchers' acquaintances. All scans of enrolled subjects were normal on visual assessment of a neuroradiologist. Subjects underwent multidimensional assessment including clinical, neurological, and neuropsychological evaluations, and drawing of a blood sample (not used for the purposes of the present study). Data coming from normal controls were used only to compute W scores in each selected perfusion ROI.

#### SPECT scan

Both patients and normal controls underwent SPECT scan in the nuclear medicine department of the Ospedali Riuniti Hospital in Bergamo. Each patient received an intravenous injection of 925 MBq of technetium- 99m ethyl cysteinate dimer (^99m^Tc-ECD) in resting conditions, lying supine with eyes closed in a quiet, dimly lit room. 40–60 min after injection, brain SPECT was performed using a dual-head rotating gamma camera (GE Elscint Helix) equipped with low energy-high resolution, parallel hole collimators. A 128 × 128 pixel matrix, zoom = 1.5, was used for image acquisition with 120 views over a 360° orbit (in 3° steps) with a pixel size and slice thickness of 2.94 mm. Butterworth filtered-back projection (order = 7, cutoff = 0.45 cycles/cm) was used for image reconstruction, and attenuation correction was performed using Chang's method (attenuation coefficient = 0.11 cm-1). Images were exported in DICOM format.

#### SPECT processing protocol

To achieve a precise normalization, we generated a study-specific SPECT template using both SPECT and MRI scans of all patients and normal controls under study, following a procedure described in detail elsewhere (Caroli et al., 2007a,b). Briefly, we created a customized high-definition MRI template, we converted SPECT scans to Analyze format using MRIcro (Rorden and Brett, 2000), and we coregistered them to their respective MRI scans with SPM2 (SPM, Statistical Parametric Mapping, version 2 (2002). London: Functional Imaging Laboratory. Available at: http://www.fil.ion.ucl.ac.uk/spm/software/spm2). We normalized each MRI to the customized MRI template through a nonlinear transformation (cutoff 25 mm), and we applied the normalization parameters to the coregistered SPECT. We obtained the customized SPECT template as the mean of all the latter normalized SPECT images. The creation of a study-specific template allows for better normalization, since low uptake in ventricular structures and cortical hypoperfusion effects are frequently present in elderly patients. For each coregistered SPECT scan, we set the origin to the anterior commissure, using the respective MRI image as a reference, and we processed all scans with SPM2 according to an optimized processing protocol described in detail elsewhere (Caroli et al., 2007a,b). Brain perfusion correlates of medial temporal lobe atrophy and white matter hyperintensities in MCI were obtained as follows: (I) we smoothed each scan with a 10 mm full width at half maximum (FWHM) Gaussian, and spatially normalized it with an affine deformation to the customized SPECT template; we applied the same deformation to the unsmoothed images; (II) we masked the unsmoothed normalized images from I to remove scalp activity using SPM2's “brainmask.” We smoothed with a 10 mm FWHM Gaussian, and warped them to the customized template with a nonlinear transformation (cutoff 25 mm); we applied the same transformation to the unsmoothed masked images; (III) we smoothed the normalized unsmoothed images from II with a 12 mm FWHM Gaussian. The following Region of Interest (ROI) were chosen for perfusion analyses in each hemisphere from the Pick atlas by a sub-routine implemented on SPM2: frontal, parietal and temporal lobes, the thalamus and the hippocampal-amygdalar complex (Maldjian et al., 2003). The choice of these regions was based on previous SPECT and PET studies in subjects with MCI (Staffen et al., 2009; Alegret et al., 2012; Yoon et al., 2012).

The whole cerebellum was chosen for normalization of ROI counts. Since perfusion values in selected ROIs did not account for age, pertinent age corrected perfusion values (hereafter called W scores), were computed in each selected ROI, following a previously published procedure (Jack et al., 1997).

#### MR imaging

Both patients and normal controls underwent brain T1-weighted MRI in the neuroradiology department of the Città di Brescia Hospital, as previously discussed (Caroli et al., 2007b). MR images were processed with SPM2 following an optimized Voxel- Based Morphometry protocol, described in detail elsewhere (Frisoni et al., 2005). Manual tracings of hippocampal and total intracranial volumes were performed using DISPLAY (DISPLAY, Brain Imaging Center—Montreal Neurological Institute. Available at: http://www.bic.mni.mcgill.ca/software). Native hippocampal volumes were normalized to the individual intracranial volumes and rescaled to the mean total intracranial volume according to the following formula ([volume/individual total intracranial volume]^*^mean total intracranial volume). Total white matter lesions load was assessed trough visual Wahlund scale on T2 and FLAIR MRI images (Wahlund et al., 2001).

#### EEG recordings

The EEG activity was recorded continuously from 19 sites by using electrodes set in an elastic cap (Electro-Cap International, Inc.) and positioned according to the 10–20 international systems (Fp1, Fp2, F7, F3, Fz, F4, F8, T3, C3, Cz, C4, T4, T5, P3, Pz, P4, T6, O1, and O2). All recordings were obtained in the morning with subjects resting comfortably. In order to keep constant the level of vigilance, an operator controlled on-line the subject and the EEG traces, alerting the subject any time there were signs of behavioral and/or EEG drowsiness. The ground electrode was placed in front of Fz. The left and right mastoids served as reference for all electrodes. The recordings were used off-line to re-reference the scalp recordings to the common average. Re-referencing was done prior to the EEG artifact detection and analysis. Data were recorded with a band-pass filter of 0.3–70 Hz, and digitized at a sampling rate of 250 Hz (BrainAmp, BrainProducts, Germany). Electrodes-skin impedance was set below 5 khz. Horizontal and vertical eye movements were detected by recording the electrooculogram (EOG). The recording lasted 5 min, with subjects with closed eyes. Longer recordings would have reduced the variability of the data, but they would also have increased the possibility of slowing of EEG oscillations due to reduced vigilance and arousal. EEG data were then analyzed and fragmented off-line in consecutive epochs of 2 s, with a frequency resolution of 0.5 Hz. The average number of epochs analyzed was 140, ranging from 130–150. The EEG epochs with ocular, muscular and other types of artifact were preliminary identified by a computerized automatic procedure (Moretti et al., 2003). Two expert electroencephalographists manually double-checked and confirmed the automatic selections. The epochs with ocular, muscular, and other types of artifacts were discarded.

#### Analysis of individual frequency bands

A digital FFT-based power spectrum analysis (Welch technique, Hanning windowing function, no phase shift) computed—ranging from 2–45 Hz—the power density of EEG rhythms with a 0.5 Hz frequency resolution. Two anchor frequencies were selected according to the literature guidelines (Klimesch, 1997, 1999), that is, the theta/alpha transition frequency (TF) and the individual alpha frequency (IAF) peak. These anchor frequencies were computed on the power spectra averaged across all recording electrodes. The TF marks the TF between the theta and alpha bands, and represents an estimate of the frequency at which the theta and alpha spectra intersect. TF was computed as the minimum power in the alpha frequency range, since our EEG recordings were performed at rest. The IAF represents the frequency with the maximum power peak within the extended alpha range (5–14 Hz). Based on TF and IAF, we estimated the frequency band range for each subject, as follows: delta from TF-4 to TF- 2, theta from TF-2 to TF, low alpha band (alpha1 and alpha2) from TF to IAF, and high alpha band (or alpha3) from IAF to IAF + 2. The alpha1 and alpha2 bands were computed for each subject as follows: alpha1 from TF to the middle point of the TF-IAF range, and alpha2 from such middle point to the IAF peak (Moretti et al., 2004, 2007a,b, 2008a,b, 2009a,b). Moreover, within theta frequency the frequency peak (individual theta frequency, ITF) was also individuated. The mean frequency range computed in MCI subjects considered as a whole are: delta 2.9–4.9 Hz; theta 4.9–6.9 Hz; alpha1 6.9–8.9 Hz; alpha2 8.9–10.9 Hz; alpha3 10.9–12.9 Hz;. Finally, in the frequency bands determined on an individual basis, we computed the relative power spectra for each subject. The relative power density for each frequency band was computed as the ratio between the absolute power and the mean power spectra from 2–45 Hz. The relative band power at each band was defined as the mean of the relative band power for each frequency bin within that band. The alpha3/alpha2 frequency power ratio was computed in all subjects. The 27 MCI patients enrolled for the present study with available MRI, SPECT, and EEG recording were finally classified as at high risk (when the alpha3/alpha 2 EEG frequency power ratio median was above 1.17) or at low risk (when the alpha3/alpha 2 EEG frequency power ratio median was under 1.17) to develop AD. The choice of this cut-off was based on the median value, in order to balance the sample size of the two groups. Moreover, it was overlapping with the value we detected in previous studies, showing an high risk to develop AD in MCI subjects with the alpha3/alpha 2 EEG frequency power ratio above the value of 1.17 (Moretti et al., 2011). Of note, this cut-off individuates a MCI group with different pattern of both hippocampal atrophy, amigdalo-hippocampal complex atrophy, and basal ganglia and thalamus gray matter lesions as compared to MCI group with alpha3/alpha2 EEG frequency power ratio values below 1.17 (Moretti et al., 2009b, 2011, 2012).

### Statistical analysis

All statistical analyses were performed using SPSS software ver. 13.0. We investigated significance of the difference between the two groups (MCI at low and at high risk to develop AD) in socio-demographic, clinical and cognitive features using χ^2^ test for categorical variables (sex, and ApoE carriers) and Student's independent *t*-test for continuous variables (volumetric, perfusion features, and EEG frequencies). In all cases we set the significant threshold at *p* < 0.05. Since native SPECT scans were coregistered to their respective MRI images, and the study-specific SPECT template was coregistered to the high-definition MRI template, all the normalized SPECT and MRI images used for the statistical analysis were coregistered to the SPM standard anatomical space. Moreover, Pearson's r correlations were assessed between the selected perfusion ROIs (in terms of age corrected W scores) and the acquired EEG frequencies in both groups.

## Results

**Figure 1:**
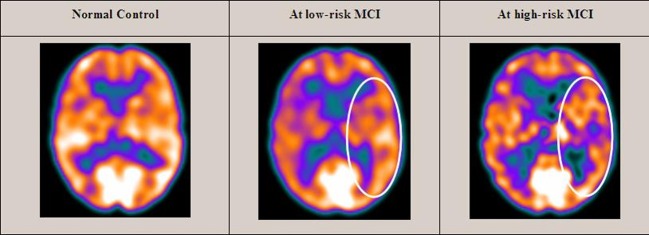
**SPECT visual rating**. The output shows a SPECT visual inspection of glucose uptake metabolism: the white square denotes an area of mild-to-moderate (purple to blue) temporparietal hypometabolism in one of the 14 at low risk and in one of the 13 at high risk MCI patient respect to one of the 17 enrolled controls.

Twenty-seven MCI patients were enrolled for the present study and they were classified as at high risk (when the a3/a2 EEG rhythm median was above 1.17) or at low risk (when the a3/a2 EEG rhythm median was under 1.17) to develop AD. The two groups (AD high risk, *N* = 13, AD low risk, *N* = 14) were similar for age (*p* = 0.56), education in years (*p* = 0.87), gender (*p* = 0.17), ApoE genotype (*p* = 0.15), MMSE scores (*p* = 0.31), and white matter lesions load (*p* = 0.88; Table 1). Figure 1 shows the visual rating scale of the SPECT scans representative of normal control, MCI with low and MCI with high risk to convert in AD, respectively. ANOVA results show that the selected cut-off was effective in detecting two different groups: patients with high risk to develop AD show significantly higher alpha3/alpha2 power ratio than patients with low risk (*p* = 0.0001). Moreover, a control analysis was performed on the the single frequencies. The results show that the increase of alpha3/alpha2 frequency power ratio was due to both increase of alpha3 (*p* = 0.001) and decrease of alpha2 (*p* = 0.0001) and not to the modification of a single frequency. This control analysis was performed because the change of only one frequency could be due to the chance. But it was not the case.

**Table 1:** **Socio-demographic, clinical, and volumetric features in MCI patients by risk to develop AD**.

	**At low-risk MCI**	**At high-risk MCI**	***p***
*N*	14	13	
Age (years) [Range]	69.1 ± 7.6 [57 ÷ 83]	70.6 ± 5.5 [62 ÷ 78]	0.555
Gender (females)	6 (43%)	9 (69%)	0.168
Education (years) [Range]	8.2 ± 4.3 [4 ÷ 18]	7.9 ± 4.5 [3 ÷ 18]	0.865
MMSE score [Range]	27.9 ± 1.6 [25 ÷ 30]	27.2 ± 1.9 [24 ÷ 29]	0.309
ApoE ε4 genotype (carriers)	2 (29%)	5 (39%)	0.152
Left hippocampal volume (mm^3^) [Range]	2606 ± 353 [1923 ÷ 3017]	2073 ± 412 [1234 ÷ 2641]	0.001
Right hippocampal volume (mm^3^) [Range]	2581 ± 473 [1549 ÷ 3150]	2296 ± 501 [1589 ÷ 3086]	141
Wahlund total score [Range]	3.58 ± 3.29 [0.0 ÷ 10.0]	3.78 ± 2.63 [0.0 ÷ 7.0]	0.886

Values are mean ± SD for continuous variables or frequency (percentage) for gender and ApoE carriers.

**Figure 2:**
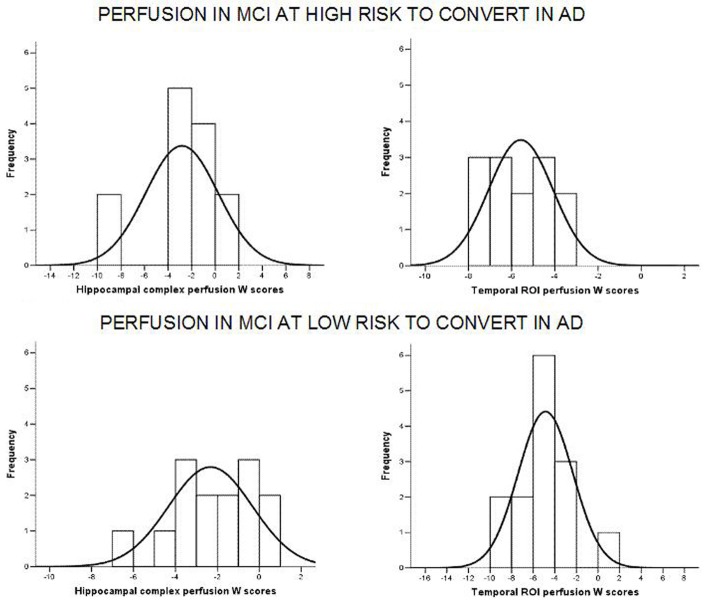
**Histograms of the perfusion W scores distribution in at low and at high risk patients for hippocampal complex (left column) and temporal (right column) ROIs**. Generally, for at high risk MCIs, perfusion scores are attested on lower values than at low risk patients. Black curve is the approximation to the normal distribution. X axis represents perfusion W scores while Y axis depicts the number of patients (frequency) for each W score.

Of note, no differences were found for beta 1, beta 2, gamma, theta EEG power, and theta/gamma frequency power ratio (all *p* > 0.11) (Table 2). Although the mean perfusion in all the selected ROIs was similar between groups (all *p* > 0.38), in the group with high alpha3/alpha2 frequency ratio there is a constant trend to a lower perfusion (see Figure 2). Moreover, left hippocampal volumes were lower for AD-high risk patients respect to low risk ones (*p* = 0.001) (Table 1). Data coming from normal controls were used only to compute W scores in each selected perfusion ROI, but their summarized socio-demographic, clinical, and volumetric features as well as their perfusion W scores can be found in Table 3.

**Table 2:** **Brain perfusion and EEG rhythms in MCI patients by risk to develop AD**.

	**At low-risk MCI**	**At high-risk MCI**	***p***
*N*	14	13	
Frontal perfusion (W scores) [Range]	1.2 ± 2.7 [−3.5 ÷ 5.5]	0.7 ± 3.6 [−3.1 ÷ 11.8]	0.707
Parietal perfusion (W scores) [Range]	2.2 ± 2.7 [−2.5 ÷ 7.0]	1.7 ± 3.8 [−2.3 ÷ 13.3]	0.698
Temporal perfusion (W scores) [Range]	−4.9 ± 2.5 [−8.9 ÷ 0.6]	−5.6 ± 1.5 [−7.8 ÷ −3.4]	0.384
Hippocampal complex perfusion (W scores) [Range]	−2.3 ± 2.0 [−6.5 ÷ 0.1]	−2.8 ± 3.1 [−9.2 ÷ 0.6]	0.616
Thalamic perfusion (W scores) [Range]	−0.5 ± 1.9 [−3.6 ÷ 4.9]	−0.6 ± 1.4 [−3.4 ÷ 2.4]	0.860
EEG alpha 1 [Range]	1.3 ± 0.1 [1.1 ÷ 1.6]	1.4 ± 0.1 [1.3 ÷ 1.5]	0.117
EEG alpha 2 [Range]	3.6 ± 0.3 [3.2 ÷ 4.1]	3.1 ± 0.2 [2.7 ÷ 3.5]	0.0001
EEG alpha 3 [Range]	3.7 ± 0.2 [3.2 ÷ 4.0]	4.0 ± 0.2 [3.7 ÷ 4.4]	0.001
EEG alpha 3/alpha 2 [Range]	1.0 ± 0.1 [0.9 ÷ 1.1]	1.3 ± 0.1 [1.2 ÷ 1.5]	0.0001
EEG beta 1 [Range]	0.5 ± 0.2 [0.3 ÷ 1.0]	0.4 ± 0.1 [0.2 ÷ 0.5]	0.175
EEG beta 2 [Range]	0.4 ± 0.1 [0.3 ÷ 0.7]	0.4 ± 0.1 [0.2 ÷ 0.5]	0.393
EEG theta [Range]	1.3 ± 0.1 [1.2 ÷ 1.5]	1.3 ± 0.1 [1.1 ÷ 1.6]	0.554
EEG gamma [Range]	1.0 ± 0.2 [0.6 ÷ 1.5]	0.8 ± 0.2 [0.6 ÷ 1.2]	0.114
EEG theta/gamma [Range]	1.4 ± 0.3 [0.8 ÷ 2.1]	1.6 ± 0.5 [1.9 ÷ 2.9]	0.120

Values are mean ± SD. Brain perfusion is always expressed as age-corrected scores (W scores).

**Table 3:** **Socio-demographic, clinical volumetric, and brain perfusion features of normal elders enrolled in the study**.

	**Normal controls**
*N*	17
Age (years) [Range]	69.6 ± 3.2 [65 ÷ 74]
Gender (females)	9 (53%)
Education (years) [Range]	9.8 ± 4.1 [5 ÷ 19]
MMSE score [Range]	27.8 ± 1.6 [24 ÷ 30]
ApoE ε4 genotype (carriers)^*^	1/12 (8%)
Left hippocampal volume (mm^3^) [Range]	2770 ± 274 [2089 ÷ 3351]
Right hippocampal volume (mm^3^) [Range]	2715 ± 221 [1881 ÷ 3139]
Frontal perfusion (W scores) [Range]	1.2 ± 0.1 [1.1 ÷ 1.3]
Parietal perfusion (W scores) [Range]	1.4 ± 0.1 [1.3 ÷ 1.5]
Temporal perfusion (W scores) [Range]	0.4 ± 0.01 [0.4 ÷ 0.5]
Hippocampal complex perfusion (W scores) [Range]	0.2 ± 0.01 [0.18 ÷ 0.21]
Thalamic perfusion (W scores) [Range]	0.5 ± 0.02 [0.49 ÷ 0.57]

Values are mean ± SD for continuous variables or frequency (percentage) for gender and ApoE carriers. Brain perfusion is always expressed as age-corrected scores (W scores).

* Missing data for 5 normal controls.

**Figure 3:**
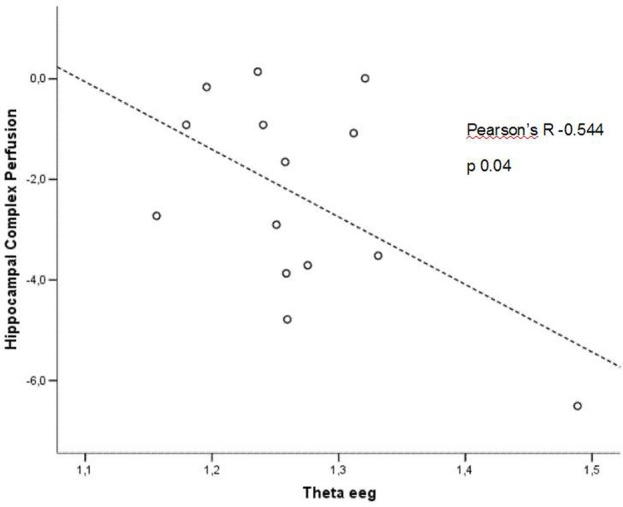
**Pearson r correlations between EEG theta rhythm and hippocampal complex perfusion in patients at low risk to develop AD**.

In patients at low risk to develop AD, significant Pearson's R negative correlation was found between perfusion in the hippocampal complex ROI and theta rhythm (*r* = −0.544, *p* = 0.044; Figure 3).

**Figure 4:**
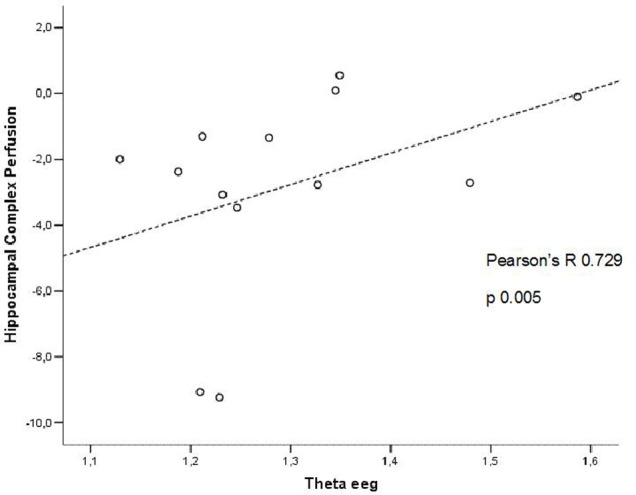
**Pearson r correlations between EEG theta rhythm and hippocampal complex perfusion in patients at high risk to develop AD**.

**Figure 5:**
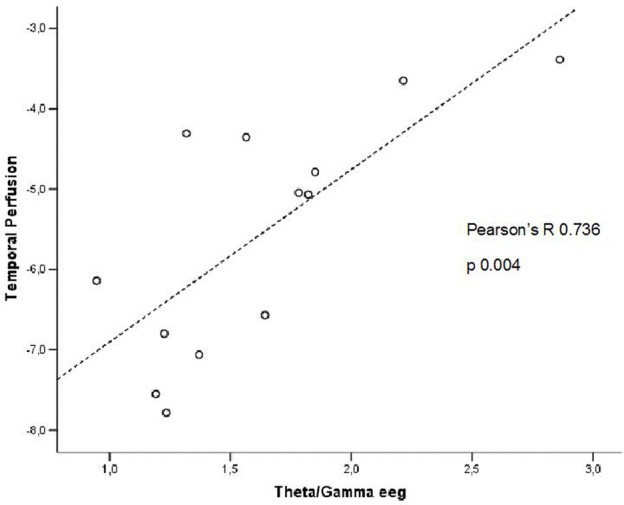
**Pearson r correlations between EEG theta/gamma frequency ratio and temporal ROI perfusion in patients at high risk to develop AD**.

In patients at high risk to develop AD otherwise, more and dissimilar correlations were found: a positive correlation, inverted respect to patients at low risk, between the perfusion in the hippocampal complex ROI and theta rhythm (*r* = 0.729, *p* = 0.005; Figure 4), while temporal ROI correlated positively with theta/gamma ratio rhythms (*r* = 0.736, *p* = 0.004; Figure 5). No other significant correlations were found in both groups between perfusion ROIs and other EEG rhythms or hippocampal volumes. Moreover, no significant correlations were found between hippocampal complex ROI and theta rhythm pooling low and high risk patients together (*r* = 0.086, *p* = 0.671).

## Discussion

The EEG alpha3/alpha2 frequency ratio in previous studies has proved useful in identifying a group at greater risk of converting in AD (Moretti et al., 2011). This group has the higher alpha3/alpha2 EEG frequency power ratio, at an orientative cut-off of about 1.17. The choice of a cut off allows the individuation of a particular population inside the group of patients with MCI. It is a very important issue of the study and makes it different from other works, usually distinguishing the MCI subjects on clinical, structural or functional aspects but not on a neurophysiological marker. The particular group individuated by the higher alpha3/alpha2 power ratio is at major risk to develope AD. The possibility to detect this risk not only in a group but also in the single patient through a cut-off is also an original contribution of this study. To be validated this EEG marker needs correlation study with morphostructural or functional milestones peculiar of AD, like as rCBF. These present results confirm that the relationship alpha3/alpha 2 identifies two distinct groups: the higher ratio characterizes a group with a smaller hippocampal volume and a constant trend of lower cerebral perfusion, even if it does not reach significance. These results confirm previous studies which have shown that patients with high risk of developing AD have left hippocampal atrophy and reduced SPECT perfusion (Frisoni et al., 2003, 2005; Frisoni, 2012). Actually, amyloid plaques deposition, NFT formation, neuronal loss, decrease in dendritic extent, and synaptic depletion are thought to disturb the communication among various cortical areas, resulting in anatomic isolation and decreased perfusion of many cortical zones (Golde, 2003). The lack of a significant difference is an obvious limitation of the work. One possible explanation is the relatively small sample size of the two groups. Given that the trend is constant, a larger sample in both groups could exploit a significant statistical difference. On the other side, it is possible that when considering two groups of patients, both with a MCI, the rCBF is not so sensible to show a large difference, as it could verify in the case of the glucose metabolism evaluation. Previous studies have demonstrated that metabolic, but not perfusional, patterns were related to severity of cognitive impairment and were more sensible in detecting prodromal MCI due to AD (Mielke et al., 1994; Forlenza et al., 2010). Further studies, with larger sample size, are mandatory to confirm these results. The present study shows a correlation between cerebral perfusion and theta rhythm. Anyway, the correlation emerges only when considering the different groups individuated on the alpha3/alpha2 frequency power ratio. This is confirmed by the finding that when the groups are merged, no correlation could be found. This is the main aspect of the study and the peculiar novelty of the results. The patients at lower risk to develop AD, who have a constant trend toward a higher brain regional blood perfusion, maintains low levels of hippocampal theta power while in patients at higher risk, with a basically lower cerebral blood perfusion, theta rhythm tends to be higher. This latter finding is also confirmed by the increased ratio of theta/ gamma frequency power ratio in the temporal region, adjacent to the hippocampus. A lot of previous studies have shown an increase of theta rhythm in patients with mild AD (Rodriguez et al., 2004, 2011), so that the increase of theta power is a robust features of AD. Theta rhythms are usually not appreciated in normal awakening EEG. However, a theta power increase is observed over the frontal and temporal areas during learning and memory tasks. The theta rhythms that are recorded during these tasks are thought to be produced by the activation of septal-hippocampal system. Hippocampus has a cholinergic innervation originating from basal forebrain, the medial septum, and the vertical limb of the diagonal band of Broca. Populations of GABAergic and glutamatergic neurons have also been described in several basal forebrain structures. The synchronized depolarization of hippocampal neurons produces field potentials that have a main frequency of 3–12 Hz and are usually known as hippocampal theta rhythm (Bland and Colom, 1993). A cholinergic-glutamatergic hypothesis of AD, in which most symptoms may be explained by cholinergic-glutamatergic deficits, has been advanced. Neuronal injury/loss may include an excitotoxic component that possibly contributes to the early cholinergic deficit. This excitotoxic component may occur, at least in part, at the septal level where somas of cholinergic neurons are found. This insult may modify septal networks and contribute to the abnormal information processing observed in AD brain, including its hyperexcitability states. According to this theory, the increased theta production in AD would derive from hyperexcitability of the septal-hippocampal system (Colom, 2006). Of note, such pattern of decreased cerebral blood flow activity and increased excitability was found even prior to the onset of cognitive impairment and cortical atrophy (Pupi et al., 2005).

On the other hand, it should be taken in mind that EEG measures electrical field variations, and a number of clinical conditions can disturb the normal electrical field of the brain. For instance, electrolyte changes may alter the appearance and time variation of the brain-generated electrical fields, and medications can slow the posterior dominant rhythm. Moreover, in assessing the frequency of the theta rhythm, cerebrovascular lesions should be considered as a possible cause of increase. By means of observations in patients with ischemic lesions, it has been suggested that delays in corticocortical fiber propagation may play a global role in determining human EEG frequencies, increasing the amount of theta activity (Thatcher et al., 1998). Increased T2 relaxation times in cortical gray matter and white matter were correlated with a shift in relative EEG power to lower frequencies in the theta range (4–7 Hz) and reduced cognitive performance (Rodriguez et al., 2011). Anyway, none of our patients suffered from acute ischemic lesions and there was no difference in the cerebrovascular load between the two groups. Moreover, the EEG frequency details of patients with chronic cerebrovascular load has been recently investigated (Moretti et al., 2007a) and they are not compatible with an high alpha3/alpha 2 frequency ratio increase. So, we are confident the our results are of neurodegenerative origin. On the whole, it emerges a picture in which it is not the simple cerebral blood perfusion rate nor a single brain rhythm that reflect the complexity of functional alteration in AD. A previous work already found that none of the regions of interest of the SPECT scans were significantly correlated with clinical severity (Müller et al., 1997).

## Conclusion

Our study reveals original and unknown aspects of a quite complex interplay between cerebral blood flow and electric activity dynamics and its association with hippocampal volume in a peculiar group of MCI patients, characterized by higher EEG alpha3/alpha2 frequency ratio.

### Conflict of interest statement

The authors declare that the research was conducted in the absence of any commercial or financial relationships that could be construed as a potential conflict of interest.
